# An Overview of Methods and Tools for Transfection of Eukaryotic Cells *in vitro*

**DOI:** 10.3389/fbioe.2021.701031

**Published:** 2021-07-20

**Authors:** Agnieszka Fus-Kujawa, Pawel Prus, Karolina Bajdak-Rusinek, Paulina Teper, Katarzyna Gawron, Agnieszka Kowalczuk, Aleksander L. Sieron

**Affiliations:** ^1^Department of Molecular Biology, Faculty of Medical Sciences in Katowice, Medical University of Silesia, Katowice, Poland; ^2^Students’ Scientific Society, Katowice, Poland; ^3^Department of Medical Genetics, Faculty of Medical Sciences in Katowice, Medical University of Silesia, Katowice, Poland; ^4^Centre of Polymer and Carbon Materials, Polish Academy of Sciences, Zabrze, Poland

**Keywords:** transfection, eukaryotic cells, polymers, viral carriers, endosomal escape

## Abstract

Transfection is a powerful analytical tool enabling studies of gene products and functions in eukaryotic cells. Successful delivery of genetic material into cells depends on DNA quantity and quality, incubation time and ratio of transfection reagent to DNA, the origin, type and the passage of transfected cells, and the presence or absence of serum in the cell culture. So far a number of transfection methods that use viruses, non-viral particles or physical factors as the nucleic acids carriers have been developed. Among non-viral carriers, the cationic polymers are proposed as the most attractive ones due to the possibility of their chemical structure modification, low toxicity and immunogenicity. In this review the delivery systems as well as physical, biological and chemical methods used for eukaryotic cells transfection are described and discussed.

## Introduction

Transfection is a widely used laboratory cell culture technique that introduces foreign nucleic acids into cells. It is a powerful analytical tool enabling study of gene functions and gene products in cells.

Nowadays, advances in life science technology enable the transfection of various types of nucleic acids into mammalian cells including: deoxyribonucleic acids (DNAs), ribonucleic acids (RNAs) as well as small, non-coding RNAs such as siRNA, shRNA, and miRNA (Borawski et al., 2007; Yamano et al., 2010; Sork et al., 2016; Shi et al., 2018).

The choice of the optimal transfection method depends on factors including the type and origin of cells and the form of introduced nucleic acids (Mirska et al., 2005; Fus-Kujawa et al., 2021). There are various strategies for introducing nucleic acids into cells that utilize variety of biological, chemical, and physical methods. The most commonly used method in clinical trials is the biological method where transfected nucleic acids are delivered to cells by viruses. The chemical transfection methods are techniques that catalyze DNA cross-membrane transport through the use of Ca^2+^phosphate, polycations or dendrimers. The physical transfection approaches include microinjection, optical transfection, biolistic transfection and electroporation (O’Brien and Lummis, 2011; Elsner and Bohne, 2017; Fus-Kujawa et al., 2021).

The location of the nucleic acid introduced into the cell is crucial to confirm effective transfection. This is also important because a major problem during transfection is nucleic acid degradation by cell nucleases. Nucleic acids, together with their carriers, are introduced into cells by endocytosis, and then these polyplexes are often trapped inside endosomes. After the maturation of such a vesicle is completed, V-ATPase, located in the endosome’s membrane, actively pumps protons into its lumen. Due to the fact that cationic polymers have a high buffer capacity, they bind protons and therefore limit the acidification of the endosome. Consequently, the proton pump pumps even more protons to keep the pH low. Transport occurs simultaneously with the penetration of chloride anions, which in turn leads to an increase in the concentration of ions and an influx of water to maintain osmolarity. The resulting osmotic pressure causes the endosome to swell, and this, combined with the polymer swelling, contributes to the endosome rupture and the release of its contents into the cytoplasm. This mechanism, known as a proton sponge, was described for cationic polymers in the late 1990s as an explanation for inducing endosomal polymer escape. The first cationic polymer investigated for the delivery of nucleic acids to cells was poly-L-lysine (Vermeulen et al., 2018). However, due to the failure of transfection with the nucleic acids alone, the search for vehicles that induce endosomal escape began. Such compounds are, for example, chloroquine or fusogenic peptides. Their drawback, however, is that they disrupt the endosome (Rehman et al., 2013). Cationic polymers having the ability to buffer the environment below the physiological pH, such as lipopolyamine and polyamidoamine, enable high transfection efficiency without the need for additional membrane damaging agents (Chen et al., 2020; Kim et al., 2020).

Importantly, transfection can be classified into two types, namely stable and transient transfection. Stable transfection refers to sustaining long-term expression of a transgene by integrating foreign DNA into the host nuclear genome. Whereas, transient transfection does not require integrating nucleic acids into the host cell genome (Kim and Eberwine, 2010; Lufino et al., 2008).

It should be taken into account that not all of these methods can be applied to all types of cells and all applications. Each method has advantages and disadvantages, thus the optimum method depends on experimental design and objective. Therefore, a wide variation is observed with respect to transfection efficiency, cell toxicity, effects on normal physiology, level of gene expression, etc. The ideal approach should be selected depending on a cell type and experimental needs, and should have high transfection efficiency, low cell toxicity, minimal effects on normal physiology, and be easy to use and reproducible.

In this publication we give an overview of both, established and emerging transfection techniques and considerations for transfection efficiency. We also discuss the technical advantages and drawbacks of their particular use. Additionally, we constructed a flow chart, in which we compiled a rough guideline to choose a gene transfer method for a particular field of application. Importantly, recent progress in transfection methods such as engineered lipid-based systems and ligand modifications for targeted delivery has been also discussed.

## Physical Methods

### Microinjection

Microinjection is a common technique that has been applied to study a variety of experimental issues in all types of cells. It is uniquely effective in transfecting cells that are difficult in processing with other methods, e.g., mesenchymal stem cells or smooth muscle cells (Stewart et al., 2016; Tiefenboeck et al., 2018).

Nuclear transfer is a method developed form a single cell microinjection used in cloning and transgenic animal generation. Nuclear manipulation includes dissecting the original nucleus from the somatic cell and transferring the desired nucleus at a single cell level. The foreign nucleus may be introduced to the host oocyte by microinjection or by fusion. The efficiency of this process is highly related to the differentiation stage of donor cells (Stein and Schindler, 2011). In comparison with reproductive cloning, nuclear transplantation produces an autologous embryonic stem cell line derived from a cloned embryo. The purpose of therapeutic cloning is to produce functional embryonic stem cells for cell replacement (Kobayashi et al., 2005).

Theoretically, in the viable and successfully transfected cells, the transduction efficiency of single-cell microinjection is nearly 100% (Zhang and Yu, 2008a). Therefore in a microinjection procedure, performed in a proper manner, injected material is the only independent variable. Another advantage of this technique is the precise dosing of injected material, as it is especially important when transducing more than one foreign materials such as proteins or peptides into a single cell (Chow et al., 2016). Moreover microinjection allows for selective delivery of the material into either the cytosol or the nucleus and it has lower cytotoxicity compared with chemical or viral transfection especially in sensitive cells like human primary neurons (Tiefenboeck et al., 2018).

Using microinjection it is not possible to inject more than 100–200 cells for each treatment. However, even in a million cell culture this number is usually enough to be statistically significant. Although, transcription analysis is possible, larger in scale protein studies can be limited by the small number of transfected cells. Proper controls are required to ensure that the injection does not affect the cell viability as the injection itself is a physical stress to cells. Additionally, when transfecting dividing cells, injected not genome integrated material is diluted with every cell division (Zhang and Yu, 2008b).

Microinjection has been commonly used in research and clinical fields such as: (1) creation of transgenic animals; (2) *in vitro* fertilization (Malter, 2016); (3) studies on transduction-challenged cells; and in (4) studies on distinguish effects of injected materials in a mixed cell cultures (Khanna, 2012; Tiefenboeck et al., 2018).

### Biolistic Method

Biolistic transfection is a mechanical method, which has potential applications in wide variety of cell and tissue types. Initially it was used for plant cells transfection as it can penetrate across cell walls. In biolistic transfection DNA or RNA is immobilized to sub-cellular size particles. Those particles are then accelerated to high velocity using a “gene gun” and then shot into cells. Therefore it does not depend on biochemical features or growth rate of the cell. Biolistic method can be used for both *in vivo* and *in vitro* cell transfections, hence it has a great potential for use in a gene therapy (Ettinger et al., 2012). This approach allows for introducing DNA or RNA to cells in tissues and currently it is the only method that allows transfection deep into tissues.

The greatest advantages of the biolistic are: ability to overcome physical barriers such as epidermis, possible multiple use in the same sample, transfecting large quantities of cells per single use, possible co-transfection of two or more DNAs in a single use, what makes it time-efficient and simple to use (Bergmann-Leitner and Leitner, 2015). Unfortunately, weakness of this method lies in the cost of the gene gun itself, although expenses associated with its utilization are low (O’Brien and Lummis, 2006).

A novel modification of biolistic method is nano-biolistic, which uses particles with smaller size about 40 nm (standard biolistic uses particles ∼1 μm in diameter). This method is as effective as standard biolistic transfection but is more appropriate for use in small cells such a HEK293 cells when examining cellular structures, and where tissue damage is a problem (O’Brien and Lummis, 2011).

By the use of high-voltage electric shocks different molecules can be introduced into cells. This procedure is called electroporation. An external electric field, which can outperform the cell membrane capacitance, may induce temporary but reversible disruption in cell membrane permeability. This temporary state of the membrane allows translocation of different molecules into the cell. The translocation consist in either simple diffusion or electrophoretical passage through the cell membrane. Electroporation was originally developed in order to be used for gene transfer, however, currently it is used with a wide variety of molecules including drugs, dyes, antibodies, oligonucleotides, etc. (Young and Dean, 2015).

Standard electroporation can be modified to affect only membranes of organelles with no effect on the cell membrane. This can be achieved with the use of extremely short pulses of very high-voltage electricity. In electroporation, the area of permeabilization of the cell membrane can be modified using different pulse amplitude (the higher amplitude is, the greater area permeabilization concerns), whereas pulse duration or number of pulses modify the degree of permeabilization. Uptake of smaller molecules does not depend on charge as those molecules are transported into the cell by diffusion (Kumar et al., 2019a). Contrary, uptake of larger molecules depends on their size, however, molecules such as dextran have been successfully loaded by electroporation (Luft and Ketteler, 2015). For successful introduction of DNA, electrophoretic forces have been shown to be important (Furuya et al., 2003).

### Laserfection/Optical Transfection

Using laser light, cellular membranes can be temporarily permeabilized what allows introducing virtually any molecule present in the surrounding medium. Based on this phenomenon various methods have been developed to use different forms of light for laserfection. Those techniques fall into two categories: in the first one only laser light is used. In the second one optical transfection is combined with chemical agents.

The mechanism, by which molecules enter into cells during laserfection still remains unclear. However, it appears that either the laser light creates a small transient hole in the cell membrane or a shock wave, produced by the laser beam absorption in the medium, induces a mechanical stress, which affects cellular membrane (Kumar et al., 2018).

Photochemical internalization, a next optical transfection method, uses photosensitive chemicals that have been encapsulated by the membranes of endocytic vesicles. When desired molecule is drawn by endocytosis, laser light is use to activate photosensitizers, inducing the formation of reactive oxygen species and destroying endosomal membrane so its content is released in the cytoplasm (Šošić et al., 2020).

Optical transfection seems to have comparable efficiency as other transfection methods. Ability to transfect single cells is a major advantage of laserfection as it is simpler than other methods with similar capability (Stevenson et al., 2010). Optical transfection has been shown to be effective in studies comparing effects of mRNA injection to the dendrite and neuronal cell bodies (Barrett et al., 2006). It has been shown that using laserfection entire transcriptom can be transferred into different cell type, that causes reprogramming of the recipient cell (Antkowiak et al., 2013).

The instrument-based methods are summarized in Table 1.

**TABLE 1:** Overview of transfection methods.

**Method**	**Cell type**	**Effectiveness**	**Cost**	**Introduced molecule**	**Advantages**	**Disadvantages**
**Instrument-based methods of transfection**						
Microinjection	Any *in vitro* cell	Close to 100%; dependant on injected material	>$1,000 Grows with process automatization level	Any (DNA, RNA, spermatozoids, proteins, peptides, drugs.)	High efficiency; Precise dosing of injected material; selective delivery; low cytotoxicity	Maximum of 100–200 cells transfected in single treatment; Laborious process
Biolistic transfection	*In vitro* and *in vivo*; e.g., primary leukocytes–lymphocytes, macrophages, and splenocytes	High	High cost of necessary equipment; low cost of utilization	DNA RNA	Possible transfection through physical barriers like epidermidis; possible cotransfection of more than one DNAs in a single use; time efficient	High cost of gene gun; Tissue damage when transfecting small cells
Electroporation	*In vitro* and *in vivo*; See above	Low to moderate	>$1,000	Plasmids; Oligonucleotides; mRNA; siRNA	High efficiency; Proven efficiency for use on tissues *in vivo*	High toxicity
Optical transfection	*In vitro* cells	Comparable to other physical methods	High cost of necessary equipment	DNA, RNA and larger objects	Ability to transfect single cells; Possible transfection with large objects	Diverse efficiency depending on technique
**Virus-based methods of transfection**						
Adenoviruses	Dividing and non-dividing cells	Expression levels are very high at the beginning, but they quickly weaken in a matter of weeks	$500–$1,000	DNA	No integration with the host cell chromosome; Easy viruses’ amplification; vectors stability in prolonged storage	Cannot induce prolonged expression; tendency to inducing a strong host immune response; Use possible only in laboratories with Biosafety Level 2 or higher
Adeno-associated virus	See above	See above	$500–$1,000	DNA	No integration with host genome; weaker immunogenicity than adenoviruses	Cannot induce prolonged expression
Retroviruses	Dividing and non-dividing cells	Stable expression	≈$1,000	RNA	Stable transfected gene expression	Possible retroviral genotoxicity
**Chemical transfection methods**						
Calcium phosphate	*In vitro* cells	High	<1,000$	DNA	Inexpensive; high efficiency; applicable to wide range of cell types; allow to transient and stable transfection	transfection efficiency is influenced by small changes of pH; consistency of precipitate
Cationic lipids	*In vitro* and *in vivo*	High	<1,000$	DNA, RNA, siRNA, and proteins	high efficiency; easy procedure; DNA, RNA and proteins may be introduced; allow to transient and stable transfection	does not work with certain cell types
DEAE-Dextran	*In vitro* cells	Moderate	<1,000$	DNA and RNA	Inexpensive; quick and easy method; wide range of cell types may be transfected; DNA and RNA may be introduced	toxicity of DEAE-dextran high concentrations; only for transient transfection; proteins may not be introduced
Magnetic beads	*In vitro* cells	High	<1,000$	DNA and RNA	simple method; high efficiency; DNA and RNA may be introduced	only adherent cells may be transfected; cells in suspension must be immobilized

## Biological Methods

### Adenoviruses

Adenoviruses are a double-stranded DNA viruses that have been used for gene delivery. This group of viruses can infect a wide range of both dividing and non-dividing cells. Generally adenoviral vectors are derived from human adenovirus serotypes 2 and 5 (Miravet et al., 2014).

Adenoviral infection begins with the attachment to the cell surface receptors and the interaction of the pontoons with α_v_β_3_ and α_v_β_5_ integrins. Subsequently, by receptor mediated endocytosis, adenovirus escapes from the endosome and heads toward the nucleus, where viral transcription and replication takes place. Completion of the infection cycle induces cell death and the release of progeny viruses. The early gene products are mostly involved in viral gene transcription, DNA replication, host immune suppression and inhibition of host cell apoptosis. The late gene products are necessary for virion assembly (Pied and Wodrich, 2019).

Upon infection adenoviral DNA is not integrated to the host cell chromosomes. Taking it into account, this method is safe but it is not possible to induce prolonged protein expression. Expression levels of the introduced genes are very high at the beginning but they quickly weaken in a matter of weeks. Additionally, adenoviruses can be amplified at high titers and are stable in prolonged storage (Wei et al., 2017). However, this method has a major drawback, which is that adenoviruses tend to induce a strong host immune response when used *in vivo* (Greber and Flatt, 2019).

**FIGURE 1:**
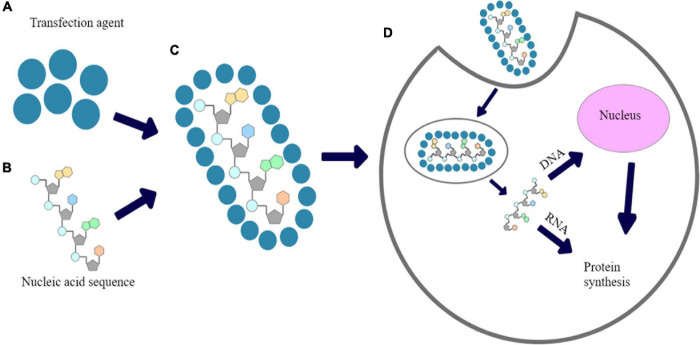
The proposed mechanism of chemical transfection: **(A)** transfection reagent; **(B)** Nucleic acid sequence; **(C)** nucleic acid polyplexed with chemical transfection reagent; **(D)** cellular uptake of polyplex and expression of introduced nucleic acid’s fragment.

Original adenoviral transcription unit limits, in view of their complexity, recombinant manipulations to non-essential for viral production regions such as E1, E2A, E3, and E4 (Figure 1). By replacing the whole adenoviral genome with exogenous sequences, a “gutless” vectors are produces. Gutless adenoviral vectors can accommodate up to 35 kb of foreign DNA. They display much lower host immunogenicity and achieve long-term expression of multiple transgenes in a single vector (Ricobaraza et al., 2020).

The generation and production of recombinant adenoviruses should be performed in a laboratory operating at Biosafety Level 2 as approved by National Institutes of Health Biosafety Committee Board^1^.

### Adenoviruses-Related Virus

Adenoviruses-related or adeno-associated virus (AAV) is a small, single-stranded DNA, non-enveloped and replication-defective virus, classified to *Parvoviridae* family. Due to its characteristic, replication of adeno-associated virus (AAV) depends on co-infections with other viruses, mainly adenoviruses (Naso et al., 2017). AAV has been shown to remain stable in a wide range of temperatures and pH, with little to none loss of activity (Rayaprolu et al., 2013).

Adeno-associated virus has been shown to be weakly immunogenic when compared to other viruses like adenoviruses, however, the capsid proteins and the nucleic acid sequence delivered can trigger immunological response. This can lead to the development of immunological memory, which may diminish the clinical efficacy of subsequent re-infections with AAV (Jeune et al., 2013).

Adeno-associated virus’s viral genome encodes three genes (rep, cap, and aap) and has a DNA size of 4 and 7 kb. Original AAV genes are commonly replaced by a transgene expression cassette in order to produce a recombinant AAV (Chen et al., 2018). Recombinant AAV, without complete viral DNA, is simply a nanoparticle designed to enter the cell and deliver its DNA cargo into the nucleus of a cell.

Recombinant episomal DNA does not integrate into the host genomes, hence it is eventually discarded over time as the cell replicates. Consequently this leads to the loss of the transgene expression, with the rate depending on the turnover rate of the transfected cell. All this indicates that recombinant adeno-associated virus could be ideal for certain gene therapy applications.

Therefore, AAV is one of the most promising vectors for gene therapy today. Due to its stability, different routes of administration and delivery strategies can be attempted. AAV can be delivered via systemic, intramuscular, CNS, cardiac or pulmonary delivery. Already many examples of clinical studies employing recombinant AAV vectors has been tried such as therapy for Leber Congenital Amaurosis, ACHM blindness, Alzheimer’s disease or hepatitis (Naso et al., 2017).

### Retroviruses

*Retroviridae* family is a group of RNA viruses that by the enzyme commonly named reverse transcriptase (DNA polymerase dependent on mRNA) are retro-transcribing their genomes into DNAs. Retro-transcribed DNA can be integrated into the genome of the host cell with present enhancers and other regulatory elements that regulate the expression of viral genes. A successful infection or, in case of vectors, transduction always leads to a stable genetic modification of the host cell (da Silva et al., 2006; Liao et al., 2017). This creates a major problem that has become a priority in retroviral development, i.e., integration of transduced genome can lead to deregulation of proto-oncogene expression and as such has long been known as a driver for retroviral genotoxicity (Biasco et al., 2017).

All retroviruses can potentially be used as a vectors, however, researchers focus mainly on four groups of vectors: lentiviruses (Human Immunodeficiency virus, HIV), gammaretroviruses (murine leukemia virus, MLV), spumaviruses (human foamy virus, HFV) and alpharetroviruses (Elsner and Bohne, 2017; Table 1).

– *Lentiviruses* (HIV) are widely used due to their ability to infect both dividing and non-dividing cells (Elsner and Bohne, 2017). The first lentiviral-based gene therapy was approved in the United Stated in August 2017 in pediatric and young adult patients treatment with acute lymphoblastic leukemia. Several additional gene therapies are currently in late-phase trials. The third generation SIN lentiviral vectors have been shown to be safe when used to transfer genes into both stem cells and T cells. In theory there is a risk of potential insertional oncogenesis with lentiviral vectors but no cases have been reported either with natural HIV or gene therapy using lentiviral vectors (Milone and O’Doherty, 2018).
– *Gammaretroviruses* (MLV) can be employed to infect cells considered as resistant to non-lentiviral vectors. They can deliver genes to non-dividing cells and potentially may be useful in gene therapy to retroviral diseases.
– *Spumaviruses* (HFV) can package large transgene cassettes and have desirable safety profile. Currently they are studied for potential use in treatment of HIV/AIDS and give a promising outlook in preclinical studies (Olszko and Trobridge, 2013).
– *Alphateroviruses* (Elsner and Bohne, 2017) such as Avian Sarcoma Leukosis Virus may be modified to produce self-inactivating vectors (SIN). They are characterized by relatively neutral integration pattern resulting in low genotoxicity. This suggest that alpharetroviruses may become an interesting alternative to other, commonly used, retroviral vectors (Suerth et al., 2012).

Differences between various viral carriers for transfection are presented in Table 2.

**TABLE 2:** The differences between the different viral vectors.

**Method**	**Advantages**	**Disadvantages**
**Virus transfection comparison**		
Adenoviruses	– Able to infect both dividing and non-diving cells – High safety when used *in vivo* due to not integrating to the host cell genome – Easy amplifying and storage – “Gutless” adenoviruses (see chapter 3.1.) can accommodate up to 35 kb of foreign DNA	– Unable to induce prolonged protein expression – Induce strong host immune response *in vivo* – Handling adenoviruses should be performed in laboratories with Biosafety Level 2
Adenoviruses-related virus	– Stability at different temperatures and pH – Lower immunogenicity when compared to adenoviruses – Does not integrate to host genome – Different routes of administration *in vivo*	– Capsid proteins and delivered nucleic acid sequence can induce immunological response – Unable to induce prolonged expression
Retroviruses	– Able to give stable expression not diminishing in time – Able to infect both dividing and non-dividing cells – *Spumaviruses*: can package large transgene cassettes and have desirable safety profile – *Alpharetroviruses*: low genotoxicity	– Can potentially cause retroviral genotoxicity – Effects of transfection are mostly irreversible

## Chemical Transfection

### Calcium Phosphates

Calcium phosphate is positively charged molecule used in order to DNA introduction into mammalian cells. It forms complex with negatively charged DNA in a form of precipitate. Complexes of DNA and calcium phosphate are added to phosphate buffer dropwise in order to proper aeration to avoid forming clumped DNA.

Endocytosis or phagocytosis are mechanisms based on which calcium phosphate transfection takes place. In this method DNA is mixed with calcium chloride. Such a transfection is proper for adherent cells and those growing in suspension but the efficiency is cell type dependent. Some cell types such a HeLa cells, are sensitive to this transfection’s method and others take DNA by endocytosis inefficiently. Unfortunately, reagent consistency is critical for reproducibility. Only small pH changes can compromise transformation efficiency. Despite this transfection method is inexpensive, it also generates mutated DNA at a high frequency (∼1% per gene). It is connected only with chromosomal DNA of the host cell. The mutations occur in a short space of time after DNA is introduced in the nucleus. These mutations are mainly base substitutions and deletions (Kumar et al., 2019b, 2020).

### Cationic Lipids

Cells transfection with cationic lipids is called lipofection or lipid-mediated/liposome transfection (Zhi et al., 2018). This technique uses a positively charged (cationic) lipid/liposomes which are amphiphilic molecules and interact electrostatistically with negatively charged (anionic) phosphate residues of DNA and cell membranes. Mechanism of introduction of complexes of cationic lipid and nucleic acid in liposome was explained by endocytosis, followed by complex release into the cytoplasm. In case of DNA, it needs to be transported into the nucleus, while the mRNA is directed and remains within the cytoplasm (Ewert et al., 2010). Cells transfection with cationic lipids is advantageous because of wide range of cell types used, from primary cells to various cell lines being adherent or cultured in suspension, as well as because of high efficiency of this method. It allows to deliver the DNA, RNA and protein of a broad range of molecular mass to the cell, and it is employed for either, transient and stable transfection (Huang et al., 2012; Paecharoenchai et al., 2012; Zhi et al., 2018).

However, the transfection efficiency with the use of cationic lipids depends on parameters such as DNA quantity and quality, the ratio of transfection reagent to DNA quantity, incubation time of lipid-DNA complex and cells density at time of complex addition, therefore the procedure optimization is recommended in the individual study conditions (Huang et al., 2012; Paecharoenchai et al., 2012; Zhi et al., 2018).

The most commonly used cationic lipid in *in vitro* systems is Lipofectamine/Lipofectamine 2000, which consists of a mixture of 2′-(1″,2″-dioleoyloxypropyldimethyl-ammonium bromide)-*N*-ethyl-6-amidospermine tetratrifluoroacetic acid salt (DOSPA) and 1,2-dioleoyl-*sn*-glycero-3-phosphatidylethanolamine (DOPE). It complexes with negatively charged nucleic acid molecules to allow them to overcome the electrostatic repulsion of the cell membrane. Additionally, Lipofectamine’s cationic lipid molecules are formulated with a neutral/helper co-lipid. Such a formulation increases transfection efficiency of plasmid DNA, mRNA, and siRNA in conditions *in vitro* in both, dividing and non-dividing cells (Dalby et al., 2004; Šimčíková et al., 2015; Zylberberg and Matosevic, 2016).

An important component of cationic lipid structure is a linker. This part is responsible for lipid biodegradability in target place as well as its stability, toxicity and transfection efficiency with liposomes. Various linkers such as ether, amido-, carbonyl-, ester-, and carbamoyl-based linkers are known. First two linkers are more stable comparing with carbaminian- and ester-based linkers (Ghosh et al., 2002). Use of amid-linked lipids enables better fusion of liposomal membrane and increased nucleic acid uptake comparing with ester bonds (Gopal et al., 2011; Zylberberg et al., 2017). Interestingly, monodisperse liposomes are an interesting approach for nucleic acids delivery. They exhibit high encapsulation efficiency that allows to design nanoliposomes encapsulating small interfering RNA (siRNA) (Matosevic and Paegel, 2011; Belliveau et al., 2012).

### DEAE-Dextran

Diethylaminoethyl-dextran (DEAE-dextran) is a cationic polymer that forms complexes with negatively charged DNA. It is used as a complexing agent for nucleic acids (DNA and RNA) and also as a coating of liposomes (Siewert et al., 2019).

Complexation of DNA with DEAE-dextran via electrostatic interactions results in positively charged complex formation that adheres with negatively charged plasma membrane. Entering of this complex into cytoplasm is possible through osmotic shock induced by dimethyl sulfoxide (DMSO) or glycerol or it crosses the plasma membrane via endocytosis. Despite this method is inexpensive and easy to perform, applied high concentration of DEAE-dextran can be toxic for transfected/treated cells. Unfortunately, DEAE-dextran can be used only for transient transfection and its efficiency varies between cell types. It is usually less than 10% in primary cells. It was demonstrated that transfection with DEAE-dextran is capable for both DNA and RNA (Kumar et al., 2018; Siewert et al., 2019).

All so far described chemical methods are listed in Table 1 and their pros and cons are summarized there.

### Magnetic Beads

Transfection that uses magnetic force/field in order to DNA introduction into cells is called magnetofection. Complexes of particles consisting of iron oxide and nucleic acid are formed by salt-induced colloidal aggregation and electrostatic interactions. This rapid, non-viral procedure may be used only for adherent cells but it is universal technology adapter for many types of nucleic acids. Suspended cells should be first immobilized or centrifuged. After addition of nucleic acid-particles complexes to the cell, they are directly transported onto magnetic plate as a source of magnetic field. It allows to increase of process efficiency and it is appropriate even if low amount of DNA is applied. Nucleic acid is taken by endocytosis and pinocytosis that allows to keep membrane architecture intact. Whole procedure may be performed in the presence of serum in cell medium that is an obstacle in different transfection methods (Santori et al., 2006; Fus-Kujawa et al., 2021).

Superparamagnetic iron oxide nanoparticles (SPIONs) have been previously analyzed as gene carriers due to their promising properties such as high stability and susceptibility to modification. These molecules use magnetic absorption in order to overcome intra- and extracellular barriers. In turn, complexes of plasmid DNA (pDNA) and small interfering RNA (siRNA) with these nanoparticles are introduced into cells because of magnetic field (Delyagina et al., 2014; Fang et al., 2016).

### Cationic Polymers (Linear Polymers, Star Polymers, and Dendrimers)

Many types of polymers were successfully tested for gene delivery and/or reproductive medicine (Nitta and Numata, 2013; Dzięgiel, 2015). One of the most common method for transfection is a use of cationic polymers that have been demonstrated as gene delivery vehicles with high solubility in aqueous solution (Bettinger et al., 2001; Yamamoto et al., 2009; Uzgun et al., 2011; Schallon et al., 2012). Because of their positive charge, they are an alternative for viral carriers in order to pDNA, mRNA or siRNA introduction into cells. In turn, DNA is negatively-charged due to presence of phosphate acid residues in its structure, thus, it forms polyplexes with cationic polymers based on electrostatic interactions. Polyplexed pDNA is prevented against degradation by cellular nucleases (Fus-Kujawa et al., 2021). A mechanism of chemical transfection is shown in Figure 1. Inefficient cellular uptake of unprotected nucleic acids arises from electrostatic interactions between anionic phosphate backbones of such polymers and the negative charge of plasma membranes. Moreover, when naked nucleic acids are directly delivered *in vivo*, they may be rapidly cut by cellular nucleases. Taking it together, a proper carriers are required in order to primarily prevent the nucleic acids against unspecific degradation. The ability of cationic polymers to induce the endosomal escape has been already explained (Lotte et al., 2018).

Cationic polymers present ability to more efficient DNA condensation in comparison to cationic lipids. Chemical structure of these macromolecules may be linear or branched (Fus-Kujawa et al., 2021). The group of branched polymers includes star-shaped macromolecules, which are of growing interest for applications in gene delivery systems, mainly due to obtaining a highly efficient delivery machinery. The unique global star structure results in a high charge density. This is an advantage during interaction with genetic materials. Also, scientific reports specified that important for the delivery systems are molar masses, architecture, degree of branching and charge density of star polymers (Wu et al., 2015). The above mentioned properties also affect the cytotoxicity (Li et al., 2010; Xiu et al., 2013).

The most popular cationic polymers are linear or branched [poly(ethylene imine)]s (PEIs), dendritic polyamidoamine (PAMAM), poly(L-lysine) and PDMAEMA [poly(*N*,*N*-dimethylaminoethyl methacrylate] (Lachelt and Wagner, 2015; Englert et al., 2018). It has been already demonstrated that cationic star polymers are good carriers for plasmid DNA (Georgiou et al., 2005; Nakayama, 2012; Mendrek et al., 2015; Rinkenauer et al., 2015; Gibson et al., 2020), siRNA (Cho et al., 2011, 2013; Schallon et al., 2012; Boyer et al., 2013; Dearnley et al., 2016) and mRNA (Yamamoto et al., 2009; Fus-Kujawa et al., 2021). Use of cationic star polymers with arms made of *N*,*N*-dimethylaminoethyl methacrylate and copolymer stars of DMAEMA and di(ethylene glycol) methyl ether methacrylate (DEGMA) for efficient DNA complexation and the transfection of human cells was proven with success (Mendrek et al., 2015). Application of stars with P(DMAEMA-co-POEGMA-OH) [poly(*N*,*N’*-dimethylaminoethyl methacrylate-co-hydroxyl-bearing oligo(ethylene glycol) methacrylate)] as the carriers of pDNA and mRNA was also documented/confirmed (Fus-Kujawa et al., 2021).

**FIGURE 2:**
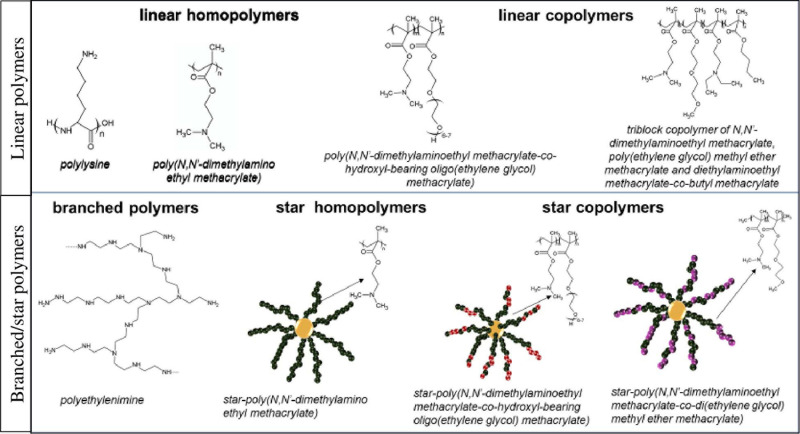
Examples of cationic polymers used in gene delivery systems.

It has been also reported that binding of mRNA to PEI, PLys, and PAMAM (Bettinger et al., 2001) is stronger/tighter than to pDNA. Linear copolymers of DMAEMA and OEGMA, PLys-co-poly(ethylene glycol), and PLys-co-poly(*N*-isopropylacrylamide) have been applied in order to obtain mRNA polyplex nanoparticles. Similarly, copolymers of DMAEMA, OEGMA, *n*-butyl methacrylate and *N*,*N*-diethylaminoethyl methacrylate elements have been already used in order to form polyplexes with mRNA (Uzgun et al., 2011; Cheng et al., 2012; Fus-Kujawa et al., 2021). Examples of the commonly used cationic polymers as gene delivery systems is presented in Figure 2.

Cytotoxicity of carriers used in cells transfection is a big problem. The perfect carrier should be non-toxic for treated cells and should allow to obtain high efficiency of the transfection. Polymer structure modifications such a pegylation allows to decrease the polymer cytotoxicity through introduction of poly(ethylene) glycol into carriers structure (Georgiou et al., 2005; Mendrek et al., 2015; Gibson et al., 2020). Pegylation also prevents non-specific interactions between carrier and cell membrane (Jiang et al., 2019).

#### Polymeric Nanoparticles

Variety of natural and synthetic polymers have been used in order to efficient delivery of nucleic acids. It has been documented that efficient and targeted delivery of antisense oligonucleotides (asODN) is possible using folic acid (FA)-conjugated hydroxypropyl-chitosan (HPCS) nanoparticles (NP). This approach allows to reduce production of P-gp and therefore, overcome drug resistance. The ability to *MDR1* gene levels inhibition *in vitro* has been documented for FAHPCS-asODNs NPs (Wang et al., 2010). The potential of these nanoparticles reveal researches that have proven the use of OX-26-transferrin-targeted poly (ethylene glycol) (PEG)-ylated immunoliposomes carrying expression plasmids of the gene-encoding tyrosine hydroxylase in model of Parkinson’s disease in rats (Zhang et al., 2003).

Interestingly, enhanced siRNA delivery has been reached by surface modification of poly(lactide-co-glycolide) PLGA NPs with polyethyleneimine (PEI) utilizing acetyl derivative (Andersen et al., 2010).

#### Nanoparticles Made of Solid Lipids

Yu et al. used Solid Lipids (SLNPs) for the targeted delivery of therapeutics to the alveolar macrophages. For this purpose, a mannan-based PE-grafted ligand has been synthetized. Then, it was used in order to prepare Man-SLN–DNA through surface modification of DNA-loaded cationic SLN. This obtained SLNP was characterized by higher gene expression in *in vivo* comparing with non-modified SLN–DNA and Lipofectamine 2000-DNA. Such a modification may be a chance for targeted gene delivery (Yu et al., 2010).

#### Inorganic Nanoparticles

Popular non-viral vectors are also organically modified silica (ORMOSIL) NPs allowing efficient delivery. Features that allow NPs to be surface-functionalized with amino groups of DNA are: high monodispersity, and stable suspension in aqueous solutions. It has been proven that it is possible to manipulate the biology of neural stem progenitor cells *in vivo*. ORMOSIL as a non-viral platform for gene delivery have a great potential for efficient therapeutic manipulation of these cells (Bharali et al., 2005).

## Targeted Delivery

Currently, many strategies are designed in order to target delivery of nucleic acids with high efficiency. These strategies concern modifications of nanoparticles with specific ligands.

Targeted liposome gene therapy has been documented as promising in clinical trials. Integral part of targeted activity of liposomes are features such as: lipids cargo to DNA cargo ratio, ester bonds, size, chain length and type of ligands complexation (Zylberberg et al., 2017). Despite PEGylation is used in order to induce endosomal escape, it causes reducing the delivery efficiency. Targeting strategies that have been documented as most promising include peptides, antibodies, aptamers and folate (Zylberberg et al., 2017). The most important problem of liposomes with targeting ligands is limited penetration efficiency in reaching the target site because of insufficient interaction between the target and targeted liposomes. This is more difficult as different cell types have different surface ligands.

Folate-linked liposomes are known as cargo delivery system based on endocytosis. Despite join of folate and liposome enables increased stability comparing with peptide conjugation, non-specific interaction with folic acid carriers may be a big problem. Currently, promising are dual-targeting approaches, that can increase specificity and the synergistic interaction between targeting antibodies and chemotherapeutics in order to provide enhanced anti-tumor effect (Qin et al., 2014).

The most popular targeting ligands for gene therapy are antibodies because of high specificity to their ligands. Nevertheless, the biggest problem of antibody-based systems is immunogenicity. Use of immunoliposomes to human cancer cells via antibody fragment has been also reported. It is a novel strategy allowing to reduce immunogenicity. Recently, a dual-targeting system has been developed as PEGylated liposomes that are modified with OX26 and peptide CTX encapsulating pC27 (Yue et al., 2014).

Interestingly, various peptides (e.g., PEI) are also known as targeting ligands because of their small size and high stability. Sequence of peptide determines immunological response and its biological activity. Importantly, hydrophobic sequences of peptides show reduced efficiency of delivery. Integration between targeting peptide and its receptor is strictly dependent on the peptide sequence and the strategy of modification. The stability of peptide-liposome complexes is lower comparing with liposomes of folate. Liposome-conjugated peptides with RGD sequence (arginine–glycine–aspartate) bind to integrins that are responsible for the adhesion of cells and extracellular matrix (Amin et al., 2015).

Cell-penetrating peptides (CPPs) are short amino acids sequences that are usually amphipathic. They contains protein transduction domains and are able to transport molecular cargo inside the cell (Nakase et al., 2012). There are at least two possible ways of CPPs entry. Possible pathways concern direct penetration, endocytosis-mediated translocation and translocation with formation of transitory membrane structure. The vast majority of known CPPs are not specific nor for cells nor tissue. They are based on positively charged amino acid sequence at physiological pH and electrostatic interactions with negatively charged surface glicoproteins (Mishra et al., 2011). Importantly, arginine-rich CPPs have the greatest potential for penetration of cells. It allows also to enhance influx of peptide to cells (Hirose et al., 2012). Interestingly, incorporation of cysteine residue to TATp increases transfection efficiency (Zylberberg et al., 2017). It has been demonstrated that TAT-mediated delivery complexed with amphipathic peptide LK15, results in improved delivery of plasmid DNA to HT29 and HT1080 cell lines (Saleh et al., 2010).

Other type of ligand for targeted delivery is short single-stranded DNA/RNA oligonucleotides, so called aptamers. Mechanism of their action is cells type-specific. Compared to antibodies, aptamers show higher recognition of target antigen and are non-immunogenic. They are also less heat-labile and can more rapidly diffuse into tissues. Importantly, these oligonucleotides tend to degrade more quickly. Aptamers bind to target by hydrogen bonds, van der Waals bonds and electrostatic interactions (Prakash and Rajamanickam, 2015).

## Transient and Stable Transfection

The choice between transient or stable transfection will usually depend on experimental goals and cell type. In short, transient transfection exerts a temporary influence on cells, whereas stable transfection leads to permanent genetic changes that are usually passed on to future cell progeny (Table 3).

**TABLE 3:** Comparison of transient and stable transfection.

**Transient transfection**	**Stable transfection**
Transfected DNA is not integrated into the genome, but remains in the nucleus	Transfected DNA integrates into the genome
Transfected genetic material is not passed onto the progeny	Transfected genetic material is carried stably from generation to generation
Does not require selection	Requires selective screening for the isolation of stable transfectants
Both DNA vectors and RNA can be used for transient transfection	Only DNA vectors can be used for stable transfection
High copy number of transfected genetic material results in high level of protein expression	Single or low copy number of stably integrated DNA results in lower level of protein expression
Generally not suitable for studies using vectors with inducible promoters	Suitable for studies using vectors with inducible promoters

### Transient Transfection

Transiently transfected cells express the foreign gene but do not integrate it into their genome. Thus the new gene will not be replicated. Nucleic acids may be transfected in the form of a plasmid (Nejepinska et al., 2012) or as oligonucleotides (Igoucheva et al., 2006).

Transient transfection often is used for studying the effects of short-term expression of genes or gene products, such as gene knockdown or silencing with inhibitory RNAs, or protein production on a small scale. Transient transfection with mRNA can deliver even more rapid results than with conventional plasmid DNA, because mRNA can be expressed outside of the nucleus; in some systems, it is possible for transfected mRNA to be expressed only minutes after transfection.

These cells express the transiently transfected gene for a finite period of time, usually several days, after which the foreign gene is lost through cell division or other factors (Kim and Eberwine, 2010).

### Stable Transfection

Stable transfection refers to sustaining long-term expression of a transgene by integrating foreign DNA into the host nuclear genome or maintaining an episomal vector in the host nucleus as an extra-chromosomal element. The hallmark of stably transfected cells is that the foreign gene becomes part of the genome and is therefore replicated. Descendants of these transfected cells, therefore, will also express the new gene, resulting in a stably transfected cell line (Lufino et al., 2008).

Stable transfection is often required for large-scale protein production, research into long-term gene regulation, the generation of stable cell lines, and for gene therapy. As the method requires successful DNA integration into the host genome, it is often much harder to achieve than transient transfection, and typically has lower transfection efficiency. Moreover, it requires selective screening and clonal isolation. Stable integration can occur randomly with plasmids, actively at random sites with help of transposases or viruses, or site-specifically when using genome editing tools like CRISPR (Recillas-Targa, 2006; Kim and Eberwine, 2010).

Summarizing, stable transfection is more useful when long-term gene expression is required. Because the stable transfection of cells is a longer and more arduous process, it is reserved for research that definitively demands it, such as protein production on a large scale, longer-term pharmacology studies, gene therapy or research on the mechanisms of long-term genetic regulation.

## Transfection Analyses

Different methods may be applied in order to assess the transfection efficiency. Direct visualization of transfected cells is possible using fluorescence microscopy when introduced molecule carries a fluorescence reporting gene. This qualitative or semi-quantitative method is fast and easy but it is impossible to distinguish signals derived from interior and exterior of cells (Peng et al., 2017). Assessment of the number of transfected cells is possible using flow cytometry. Antibodies for flow cytometry must be fluorescently labeled or they are visualized by binding to a secondary antibody. Each antibody is attached to different antigen allowing detection of transfected cells. Unfortunately, this method is expensive and time-consuming (Wu et al., 2012; Homann et al., 2017).

Western Blot method is used for quantitative or semi-quantitative analysis of proteins in transfected cells. β-actin (Jordan, 2007) and GAPDH (Dittmer and Dittmer, 2006) have been demonstrated as an internal control. Despite this method allows to simultaneous assessment of downstream protein targets regulation, it is also time consuming and non-specific protein binding may occur (Bass et al., 2017; Shi et al., 2018).

In turn, reporter genes are the perfect tool for transfection results analysis and their expression can be easily monitored. Usage of reporter genes such green fluorescent protein (GFP), luciferase and β-galactosidase has been demonstrated to be very useful (Mülhardt and Beese, 2007; Smale, 2008; Masser et al., 2016; Mendrek et al., 2018; Fus-Kujawa et al., 2021). Moreover, these reporter genes may be also used for standardization of transfection for its efficiency based on expression levels of their products. There are two ways of reporter genes usage. These genes may be used alone or fused with the gene of interest in order to assess the protein expression level, positive cells number or location of the protein of interest (Horibe et al., 2014).

Other method of transfection efficiency assessment is Real Time PCR (qPCR). It allows to direct quantitative measurement of nuclei acids level in transfected cells. Importantly, in case of transient transfection it is necessary to monitor the efficiency after each transfection round/set (Werling et al., 2015).

## Discussion

Over the past several decades, great advances in transfection methods and carriers of genetic material has been made. The transfection is the basic method of molecular analyses enabling efficient introduction of nucleic acid and transgene expression. Effective introduction of nucleic acids coding defined sequences is a great perspective for further use in gene therapy.

Transfection efficiency depends on the transfection method but also on a quality of biological material. Most importantly, cell culture should be free from any infections such bacteria (e.g., mycoplasma), funguses, viruses and chemical toxins. When cell culture is infected, results of experiment repeats can vary between each other, although applied transfection conditions are the same (Evanko, 2013). One of the main cell culture problems is contamination with bacteria classified to Mycoplasma family of which the most dangerous are: *Mycoplasma pneumoniae*, *Mycoplasma genitalium*, *Mycoplasma hominis*, *Ureaplasma urealyticum*, and *Ureaplasma parvum*. They all cause a lot of problems, and since they lack a cell wall, many antibiotics that kill bacteria by weakening their wall (e.g., penicillin) are not effective in this case. Additionally, the size of mycoplasma is in the range of 0.15–0.3 μm. On the other hand, the most popular filters used for cell culture media are 0.22 μm in pores size. Cells do not die because of mycoplasma but their metabolism can be altered leading to chromosomal aberrations, decrease in the growth rate and detachment of cells from culture surface. Mycoplasma detection methods are based on commercial kits utilizing PCR reaction and DAPI or Hoechst staining (Gopalkrishna et al., 2007; Armstrong et al., 2010; Philippeos et al., 2012; Uphoff et al., 2012; Faison et al., 2020). It is very easy to infect cells by carrying mycoplasma bacteria on our skin, with infected reagents (e.g., serum) or other infected cell culture, thus Good Laboratory Practice (GLP) should be introduced in every experimental laboratory carrying cell culture work. Mycoplasma is important not only in transfection assays but it should be taken into account before starting an experiment.

Apart from mycoplasma, cell culture may be also infected with endotoxins that may be in complexes with lipopolysaccharides (LPSs). This component builds outer membrane of most gram-negative bacteria, which may release an endotoxin to their environment when they grow actively but large amounts are released when bacteria die. Unfortunately, endotoxins are heat resistant and are able to form aggregates. Endotoxin alter cell growth and due to their hydrophobic properties they have affinity for hydrophobic materials such plastic vessels for cell culture (Lai, 2017; Nomura et al., 2017). The most commonly test used for the detection of endotoxins is the Limulus Amebocyte Lysate (LAL) test due to its reaction with lipopolysaccharides (Gnauck et al., 2016; Amidou, 2017).

An important factor influencing cell transfection, particularly of primary cells, is the cell passages number, which should be as low as possible. Cells with different number of passages may not to be equally sensitive responding to the transfection conditions (Bhuiyan et al., 2004). Additionally, genetic material used for transfection should be of high quality, purity, integrity and concentration. In case of DNA it usually is suspended in sterile water or TE buffer to a final concentration of 0.2–1 mg/ml. DNA isolation should be performed carefully in order to avoid nucleic acid contamination, i.e., with reagents used for isolation procedure such as ethanol. It is necessary to pay attention on nucleic acid ratios absorbance at A260/A280 and A260/A230 used as a measure of nucleic acids purity. The maximum absorbance for nucleic acids is at wave length 260 nm. An ideal value of A260/A280 ratio for DNA is around 1.8 and for RNA around 2.0. The value below 1.8 means contamination of the nucleic acid preparation with proteins. A260/A230 ratio is an indicator of organic contamination with reagents such phenol, chaotropic salts, ethanol, and trizol, etc. Proper range of A260/A230 value is in the range of 1.8–2.0.

Advanced transfection methods allowing efficient nucleic acids introduction to cells for expression of correct product or to fix detected mutation for gene sequence correction are the two main goals of gene therapy. Many factors affect the choice of cells transfection method. Systems utilizing viral vectors are controversial because the vector can interfere with the host’s genome integrity, thus generate unpredictable changes in genomic DNA sequence. An efficient transfection with retroviruses causes modification of the host cells (da Silva et al., 2006). Virus integration into host genome may lead to change the level of gene expression that is dangerous for transfected cell and whole organism. Importantly, immunogenicity displays a huge problem when introducing foreign nucleic acid into a eukaryotic cell. Especially adenoviruses have tendency to induce immune response of host *in vivo*. Despite adeno-associated viruses have been reported to be weakly immunogenic comparing with other viral carriers, both capsid proteins and sequences encoded by delivered nucleic acid may generate response of immune system (Jeune et al., 2013).

Therefore, non-viral carriers are perfect candidates for nucleic acids delivery. Their use do not arouse controversies because they do not integrate into host genomes and are safe for the patients. However, it has been demonstrated that many of the chemical systems may be toxic for most cells. On the horizon are new generations of carriers based on synthetic polymers, which can be well characterized and they do not contain any unknown biological impurities. One promising group of such polymers is called star polymers. Importantly, many modifications of star polymers structure have been already proved to be successful in cells transfection. These nanoparticles are highly soluble in the solution. Using these polymers as non-viral carriers for cells transfection is valuable since introduction of some chemical modifications such a pegylation, allows to obtain non-toxic polymers with enhanced effectiveness. Introduction of poly(ethylene) glycol into structure of star polymers enables to decrease their cytotoxicity (Georgiou et al., 2005; Mendrek et al., 2015; Gibson et al., 2020). Except for reducing polymers’ cytotoxicity, such structure’s modifications eliminate some non-specific interactions that may occur between genetic material’s carrier and the cell membrane (Jiang et al., 2019).

Recent progress has been made in the field of ligands modifications for targeted delivery in order to increase efficiency of transfection. Despite antibodies are the most popular ligands for targeted delivery, they may also be immunogenic. Some improvements have been made in order to decrease immunogenicity such a using of immunoliposomes through fragment of antibody. Interestingly, due to the ability of CPP such TAT to penetration of the cell, they enable to increase transfection efficiency. Taking it into account, they may also be used as targeting ligands (Saleh et al., 2010).

Nevertheless, the critical limitations of efficient gene delivery include variety of barriers. The most common are: off-target effects, endosomal escape and poor stability. Targeting ligands are able to decrease the risk of non-specific binding and promote the target cell interactions. Nevertheless, the presence of targeting moiety is not a certainty for efficient transfection. Factors such as communication between ligand and receptor and stability of nucleic acids’ carrier must be taken into account in order to enhance the targeted delivery.

## Author Contributions

AF-K, KB-R, and ALS: conceptualization. AF-K and PP: data curation. ALS: formal analysis. PT and AF-K: N/A–review and investigation. AF-K, KG, and ALS: methodology, N A–review, and project administration. ALS and AK: resources, N/A–review, software, and supervision. AF-K: validation. AF-K, KB-R, and PP: visualization. AF-K, PP, and ALS: writing—original draft. AF-K, KG, KB-R, and ALS: writing—review and editing. All authors contributed to the article and approved the submitted version.

## Conflict of Interest

The authors declare that the research was conducted in the absence of any commercial or financial relationships that could be construed as a potential conflict of interest.

## Footnotes

1 https://www.cdc.gov/labs/BMBL.html
